# Predictors for Intravenous Immunoglobulin Resistance in Patients with Kawasaki Disease

**DOI:** 10.1155/2022/2726686

**Published:** 2022-08-03

**Authors:** Wei Li, Li Zhang, Zhouping Wang, Xiufang He, Huimei Lin, Yanfei Wang, Jia Yuan, Xiaofei Xie, Xu Zhang, Youzhen Qin, Ping Huang

**Affiliations:** ^1^Department of Cardiology, Guangzhou Women and Children's Medical Center, Guangzhou Medical University, Guangzhou 510120, China; ^2^Department of Pediatric Cardiovascular, The First Affiliated Hospital of Sun Yat-sen University, Guangzhou 510080, China; ^3^Department of Medical Record, Guangzhou Women and Children's Medical Center, Guangzhou Medical University, Guangzhou 510120, China

## Abstract

**Background:**

Between 10 and 20% of Kawasaki disease (KD) patients are resistant to treatment with initial intravenous immunoglobulin (IVIG) and have a high risk of developing coronary artery lesions. Some studies have been conducted to identify predictive factors. However, the results are controversial. This study aims to identify the risk factors for IVIG-resistant KD patients in a Chinese population.

**Methods:**

We performed a retrospective analysis of medical records of consecutive KD patients from two medical centers in South China from January 2015 to December 2017. A total of 1281 KD patients were eligible for inclusion in this study and maintained follow-up for over 12 months. The KD patients were divided into two groups based on IVIG response. Clinical characteristics and laboratory variables were compared between the two groups. Multivariate logistic regression analysis was performed to identify the risk factors of IVIG resistance in KD patients.

**Results:**

Of the 1281 KD patients, 141 (11.0%) cases who were IVIG resistant to adjunctive therapies for primary treatment were classified as group 1. The remaining patients were in group 2 (*n* = 1140), classified as the control group. There was a signiﬁcant difference in male to female ratio and the length of hospital stay between the two groups (*P* < 0.05). Group 1 had a higher white blood cell count (*P*=0.01) and C-reactive protein level (*P* < 0.01) before IVIG treatment than in group 2. Group 1 had a significantly higher white blood cell count and percentage of neutrophils after the IVIG infusion than in group 2 (*P* < 0.001). In addition, the mean values of C-reactive protein level and neutrophil percentage before and after treatment difference comparison were significantly different. Multivariate analysis showed that patients presenting with coronary artery lesions in the acute phase and a C-reactive protein level >100 mg/L at diagnosis were associated with IVIG resistance in KD. During the 12-month follow-up period, group 1 had an obviously higher incidence of coronary artery lesions than group 2, and the difference between the groups was statistically significant (*P* < 0.001).

**Conclusions:**

Patients presenting with coronary artery lesions in the acute phase and elevated C-reactive protein levels before IVIG treatment might be a useful and important value for predicting IVIG resistance in KD. Risk assessment based on coronary artery lesions and C-reactive protein levels prior to the treatment may improve the outcome of IVIG resistance.

## 1. Introduction

Kawasaki disease (KD) is a systemic vasculitis of unknown etiology in children less than 5 years old that causes coronary artery lesions (CALs) in about 25% of untreated patients [1, 2]. CALs including coronary artery aneurysms or dilatations are the most serious complication of KD, which is the leading cause of acquired heart disease in children of developed countries, nowadays [3]. It has been described in all ethnicities worldwide. However, it is more common in Asian countries [4, 5]. The incidence of CALs is markedly decreased with the use of intravenous immunoglobulin (IVIG) therapy. The efficacy of high-dose IVIG administered in the acute phase of KD is well established to reduce the prevalence of CALs from about 25% to as low as 5% [3]. However, approximately 10% to 20% of patients with KD who do not respond to the initial IVIG treatment and have persisting fever and inflammation are termed IVIG resistance [6–8]. It is reported that those with IVIG-resistant KD have a higher risk of developing CALs [9–11].

There was not enough robust evidence to guide pediatricians in the recognition and diagnosis of IVIG resistance in children with KD [12, 13]. We aimed to explore potential predictors through the clinical data of patients with IVIG resistance.

## 2. Methods

### 2.1. Patients and Data Collection

We performed a retrospective chart review of medical records of children diagnosed with KD from January 2015 to December 2017 in two medical centers in South China. KD was diagnosed according to the recommended universal KD criteria proposed by the American Heart Association guidelines [14]. Patients were diagnosed with KD by at least two experienced pediatricians. The inclusion criteria of KD subjects were as follows: (1) patients were ≤18 years old, and only complete or incomplete KD cases were included; (2) patients had initial onset of KD; (3) patients received initial IVIG treatment with a 2 g/kg single infusion; and (4) patients maintained follow-up for at least 12 months. Exclusion criteria for this study included (1) patients who received IVIG treatment after 14 days of illness onset, (2) patients who had serious infections, other vascular, allergic, autoimmune, and genetic diseases, and (3) patients who had other illnesses with similar features known to mimic KD. IVIG-resistant KD patients [3] were termed as (1) patients with KD who develop recrudescent or persistent fever for at least 36 hours after the end of their IVIG infusion; (2) return of fever and 1 or more of the initial symptoms that led to the diagnosis of KD within 2 to 7 days after initial IVIG treatment. Initial treatment protocol in our study included patients treated with IVIG 2 g/kg per day as soon as the diagnosis of KD was carried out and aspirin 30–50 mg/kg per day during the acute phase until the child had been afebrile for 48 to 72 hours, followed by aspirin at 3–5 mg/kg per day for 6–8 weeks after the onset of illness or until the resolution of coronary artery abnormalities. An alternative antiplatelet medication was selected when aspirin was contraindicated [15]. Cessation of fever was defined as a body temperature of <37.5°C for 24 hours. The first day of the illness was defined as the first day of fever onset.

Information on demographics, clinical course, laboratory data, and echocardiography results were extracted from the medical records. The following data included months of age, gender, duration of fever, illness days with initial IVIG administration, and length of hospital stay. Blood samples were collected during the acute febrile phase prior to IVIG treatment and initial standard treatment after 24 hours and assessed for laboratory parameters including white blood cell count (WBC), percentage of neutrophils, C-reactive protein (CRP) levels, and the mean difference of each variable between the two groups was also compared. The mean difference was defined as follows: mean difference = laboratory parameters prior to IVIG treatment – variables 24 hours after IVIG treatment, coronary arteries based on the maximal internal diameters of the right coronary artery and the left anterior descending artery measured by echocardiography at the time of diagnosis and regular follow-up [3]. CALs were reported based on two-dimensional echocardiography with *Z* scores in this study [3, 16]: dilation: 2 to <2.5, or if initially <2, a decrease in *Z* score during follow-up ≥1; small aneurysm: ≥2.5 to <5; medium aneurysm: ≥5 to <10; absolute dimension <8 mm; giant aneurysm: ≥10; or absolute dimension ≥8 mm.

The study group was divided into two groups in accordance with their response to the standard IVIG therapy for KD patients. Group 1 included patients who received rescue therapy due to failure of the initial IVIG treatment. Patients who received a single dose of IVIG treatment and responded positively were classified as group 2. All subjects provided written informed consent signed by their parents or guardians. This study was reviewed and approved by the two participating centers' institutional review boards.

### 2.2. Statistical Analysis

A descriptive analysis was performed on all the data. Data were presented as either mean ± SD for continuous variables or as the number and percentages (%) of patients for categorical variables. The *t*-tests were used to compare normally distributed continuous laboratory values. Categorical variables were compared by a 2-sided chi-square test and Fisher exact test between the 2 groups. Univariate analysis was performed to identify the variables, and multivariate logistic regression analysis was used to identify independent predictors of IVIG-resistant KD. Results were expressed as an odds ratio (OR) with a 95% confidence interval (CI). All tests were two-sided and *P* < 0.05 was considered significant. All statistical tests were conducted with the Statistical Package for the Social Sciences (SPSS) version 21.0 (SPSS Inc., Chicago, IL, USA).

## 3. Results

### 3.1. Population Characteristics

**Figure 1:**
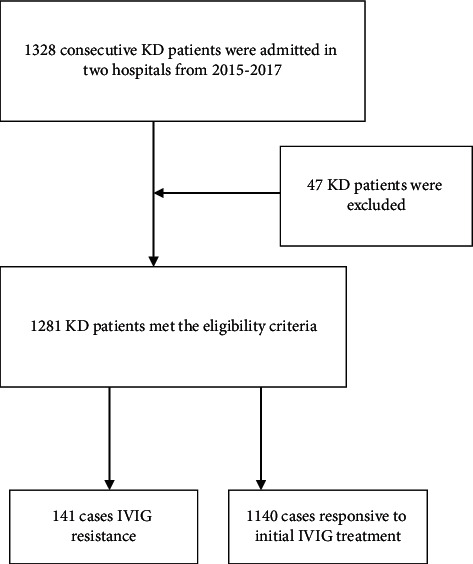
Flow chart for selection of patients with Kawasaki disease in this study. After the exclusion of patients with an inability to follow-up for 12 months, 1281 patients were eligible for the study. KD, Kawasaki disease; IVIG, intravenous immunoglobulin.

The demographic characteristics of patients are summarized in Table 1. We reviewed the clinical records of 1328 consecutive KD patients from 2 medical centers in South China. 47 patients were excluded because they failed to finish a follow-up of 12 months. Consequently, 1281 patients met the eligibility criteria and were recruited for analysis. Among the study population, 141 (11.0%) cases were defined as IVIG-resistant KD patients. All patients were classified into 2 groups. Group 1 (*n* = 141) were classified as IVIG-resistant KD patients, including 103 males and 38 females, and the male-to-female ratio of group 1 was 2.71 : 1. Patients in group 2 (*n* = 1140) were classified as controls, whereas the ratio of group 2 was 1.67 : 1 (Figure 1). Male patients were more significant in group 1 than those in group 2 (*P*=0.014). The suggested male patients were at a significantly higher risk of resistance to initial IVIG than in female patients. The mean age of group 1 was 12 months (range, 8–36 months). The mean age of group 2 was 12 months (range 11–36 months), which was not significantly different when compared to group 1 (*P*=0.746). The hospitalization day in group 1 was higher than that in group 2 (*P* < 0.05). Additionally, the duration of fever and initial administration of IVIG in group 2 was less than that in group 1, and there was a statistical significance between the two groups (*P* < 0.05). All IVIG-resistant KD children were treated with an additional IVIG dose of 2 g/kg, except one patient. In addition, 17 children were treated with corticosteroids after additional IVIG treatment because of recurrent fever. Additional IVIG treatments were effective in 87.9% (123/140) of the patients in group 1.

As shown in Table 2, the following laboratory data were obtained before and after (24 hours) of IVIG treatment. As for the laboratory parameters, group 1 had a higher WBC count (*P*=0.01) and CRP level (*P* < 0.001) than group 2 prior to IVIG treatment. Group 1 had a significantly higher white blood cell count and percentage of neutrophils after the IVIG infusion than group 2 (*P* < 0.001). In addition, the CRP level and percentage of neutrophils before and after the treatment difference in comparison were significantly different between the two groups. However, there was no statistically significant difference in comparison with WBC, percentage of neutrophils before therapy, and CRP after treatment. These results indicate that KD patients with IVIG resistance have ongoing systemic inflammation as manifested by the elevation of WBC and CRP levels prior to IVIG treatment.

We used logistic regression to compare the incidence of KD patients with IVIG resistance and other parameters, including age, gender, duration of fever, initial administration of IVIG treatment, CRP, WBC count, % neutrophils before treatment, and CAL at diagnosis, were used as covariates to control confounding factors. The variables identified by a univariable analysis to be significant predictors of IVIG resistance were as follows: gender, CRP level, and CAL at diagnosis. These 3 variables were included in the logistic regression analysis. Group 1 and group 2 comprised 48.2% and 31% of the total number of patients with CRP levels >100 mg/L before therapy, respectively. The incidence of CALs prior to IVIG treatment in group 1 and group 2 was 43.3% and 24.4%, respectively. Multivariate logistic regression analysis showed that CRP levels >100 mg/Land CAL prior to IVIG treatment were independent predictors for KD patients with IVIG resistance (Table 3).

**Figure 2:**
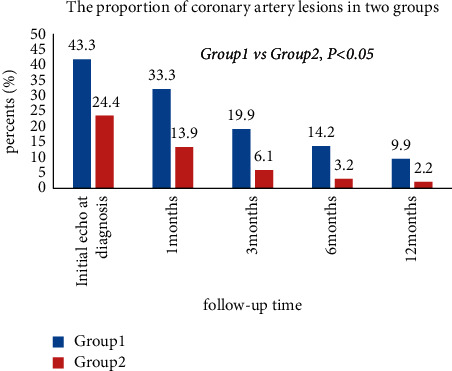
The proportion of coronary artery lesions in the two groups during the 12-month follow-up.

The number and proportion of KD patients with CALs are shown in Table 4. The proportions of CALs were compared between the two groups. The incidence rate of CALs in IVIG-resistant children was 43.3%, which was significantly higher than that of IVIG-responsive children (24.4%; *χ*^2^ = 22.975; *P* < 0.00), and there was also a higher incidence of CALs in group 1 and a statistically significant difference during 1 month, 3 months, 6 months, and 12 months of follow-up (*P* < 0.001). As shown in Figure 2, IVIG resistance was strongly associated with an increased CAL rate. Group 1 was about 2.5 times more likely to develop CALs than group 2 at 1-month follow-up. At the end of the 12-month follow-up, group 1 was about 4.5 times more likely to develop CALs than group 2. It is indicated that patients who respond to initial IVIG had a lower incidence of coronary artery abnormalities defined by Z scores than IVIG treatment resistance.

## 4. Discussion

In our study, we revealed clinical characteristics and midterm outcomes in a group of 1281 Chinese pediatric patients with KD at two centers. Our 141 cases of IVIG-resistant KD represented 11.0% of patients with KD during the study period. Our data suggest that there may be a higher risk of developing coronary artery abnormalities in IVIG-resistant KD patients. Furthermore, these results indicate that CRP >100 mg/L and CALs at diagnosis were independent risk factors of KD patients with IVIG resistance.

The incidence of patients with KD who do not respond to initial treatment with IVIG is approximately 10%–20% [17–19]. Some research studies suggest that the incidence of CALs in IVIG-resistant KD is as high as 22%–45.8% [20–23]. Our study showed that the incidence of IVIG resistance in KD is 11.0%, and there was an incidence of 33.3% in CALs in one month during the clinic visit. There were about 4.5 times more likely to develop coronary artery lesions in KD patients with IVIG resistance than with IVIG sensitivity. Male predominance may reflect a higher risk of coronary artery involvement in IVIG-resistant KD patients. This was similar to the results of previous studies [8, 9]. There was a significant difference in the time from fever onset to diagnosis and initial IVIG treatment between the two groups. Prolonged fever has been recognized as the strongest predictor of CALs [24]. Our results also showed that prolonged fever duration, fever duration before treatment, and hospitalization days occurred in IVIG-resistant patients. Previous studies also have found that there was an association with a significantly longer duration of fever and higher coronary artery involvement, suggesting that there was an excessive inflammatory response in IVIG-resistant children [25]. Because the incidence of CALs is so high, it is important to find a more beneficial treatment for these children. Previous studies showed that additional IVIG, steroid, or infliximab treatments were used for IVIG-resistant patients [1, 3]. Pediatricians must pay more attention to these high-risk patients, and the use of additional therapies in all patients in the acute phase of KD is necessary. Our study also found that additional IVIG treatment is effective in 87.9% of KD patients with IVIG resistance. It is reported to identify these patients who might benefit from more aggressive initial therapy. However, these treatments would need to be further evaluated by randomized controlled trials [26, 27].

The main pathology of KD is a systemic vasculitis of the median and small blood vessels, as evident from the high levels of inflammatory parameters like CRP, WBC count, and the percentage of neutrophils found in the acute phase [3]. Actually, the elevated WBC, CRP, and leukocytosis reflecting the vascular inflammatory response during the acute stage of illness could provide support for a diagnosis of incomplete KD clinical experience. In addition, there are some studies that have been conducted in different countries to predict the risk factors of IVIG-resistant KD. Several Japanese scholars such as Egami et al. [8], Kobayashi et al. [9], and Sano et al. [22] summarized the prediction of IVIG-resistant KD and proposed the formulation of different scoring systems comprising related clinical and laboratory findings. However, these scoring systems were inaccurate in a recent North American cohort [28, 29]. Afterward, Chinese scholar Fu et al. [11] created an alternative scoring system based on a single center research result. These prediction systems lack unity. It was suggested that these subtle genetic differences affect the performance of these prediction tools [30]. In line with previous studies [9], a significant difference was found for elevated WBC counts and CRP before IVIG therapy between the two groups. In line with the previous studies, our results also showed significantly higher levels of these markers in patients who developed CALs and resistance to IVIG. It is challenging to make a diagnosis of IVIG-resistant KD, particularly before the initial administration of IVIG. We still considered other different diagnoses of fever etiologies, particularly systemic-onset juvenile idiopathic arthritis (so-JIA). On the one hand, Hartas et al. [31] demonstrated that patients with KD who also have acute arthritis are at high risk for being IVIG-resistant. On the other hand, Dong et al. reported that so-JIA within 6 months after treatment for presumed KD, which was associated with Caucasian race, macrophage activation syndrome, and an incomplete KD phenotype [32]. Similarly, Sahin et al. proposed that IL-18 has been a useful marker in differentiating so-JIA from incomplete KD patients except for clinical manifestations and laboratory markers of so-JIA [33]. In contrast to previous studies, Ram Krishna et al. [25] found that clinical features cannot predict IVIG-resistant Kawasaki disease. The evidence suggests that IVIG-resistant KD patients with prolonged fever can have different clinical features. Therefore, we did not include this factor of clinical features in this study. The results of our study suggest that patients presenting with CALs at diagnosis and CRP >100 mg/L are independent risk factors for KD patients with IVIG resistance. Meanwhile, persistant fever and clearly increased CRP have been known to reflect the extent of systemic inflammation and vasculitis in KD patients [34–36]. Therefore, KD patients with IVIG resistance are at higher risk for CALs. We found the risk factors for IVIG resistance by two parameters in our study population. This may be used as a new novel marker for the prediction of KD patients with resistance to IVIG.

There is strong evidence that the probability of IVIG-resistant KD patients also having CALs is greater than that for IVIG-sensitive patients [26]. If KD patients with IVIG resistance could be detected and appropriately treated before additional IVIG treatment, the probability of damage to the coronary arteries would decrease, as well as the total hospitalization time [37, 38]. CALs were noted in as many as 43.3% of IVIG-resistant patients in our cohort, and IVIG resistance had a higher proportion of CALs during midterm follow-up based on the present study. So, there may be an urgent need to construct and evaluate a new novel marker to help decision-making for IVIG-resistant patients. This study aimed to explore the risk factors associated with IVIG-resistant KD and would provide evidence for treatment regimens in these patients. Those patients with IVIG resistance might be improved by combined treatment therapy or rescue therapy to reduce CALs [39–41]. It is reported that speckle-tracking echocardiography was a useful echocardiography technique for the evaluation of myocardial deformation or strain in KD patients. Previous studies [42] have demonstrated that children in the convalescent/chronic phase of KD have a subtle decrease in strain rate when compared to the acute phase, although within the normal range. This decrease is more pronounced in children with CAAs than in those without CAAs. Similarly, Dedeoglu et al. found that speckle-tracking echocardiography could evaluate impairment of left ventricular mechanics that arises especially within the left anterior descending artery territories [43]. Therefore, it is worth noting that speckle-tracking echocardiography may be helpful in predicting the resistance in patients with KD because of the higher rate of coronary artery involvement. Investigations may help to select the appropriate treatment and alleviate complications in IVIG-resistant KD patients in the future.

Although this study is a large study to be performed on Chinese children with IVIG-resistant KD in the south of China, certain limitations should be acknowledged. First, this study was a retrospective study performed by analyzing medical records. Second, the selection of control patients was based on treatment and continuous follow-up, inevitably introducing selection bias. Because of the limitations of this retrospective study, future prospective studies are needed. Despite these limitations, this study has implications for clinical practice.

## 5. Conclusions

Patients presenting with coronary artery lesions in the acute phase and elevated C-reactive protein levels before IVIG treatment might be a useful and important value for predicting IVIG-resistant KD patients. Risk assessment based on coronary artery lesions and C-reactive protein levels prior to the treatment may improve the outcome of IVIG resistance. Future prospective studies are needed to establish the true efficacy of this finding.

## Figures and Tables

**Table 1:** Demographic comparison of 1281 patients with Kawasaki disease between the two groups.

Variables	Group 1 (*n* = 141)	Group 2 (*n* = 1140)	*P* value
Age, months	12 (8–36)	12 (11–36)	0.746
≤12 months, *n* (%)	84 (59.6)	633 (55.5)	—
>12 months, *n* (%)	57 (40.4)	507 (44.5)	—
Sex (male: female ratio)	2.71	1.67	0.014
Duration of fever (days)	6 (5–9)	6 (5–7)	0.046
Initial administration of IVIG (days)	6 (5–9)	6 (5–8)	0.043
Length of hospital stay (days)	5 (3–8)	2 (2–3	<0.001

Values are expressed as median (interquartile range) or *n* (%); IVIG = intravenous immunoglobulin.

**Table 2:** Laboratory data comparison between the two groups.

Variables		Group 1 (*n* = 141)	Group 2 (*n* = 1140)	*P* value
WBC (10^9^/L)	Before therapy	17.65 ± 8.63	15.52 ± 5.50	0.01
	After therapy	12.40 ± 5.84	9.65 ± 4.00	<0.001
	△WBC	5.25 ± 8.55	6.05 ± 5.35	0.282

CRP (mg/L)	Before therapy	102.86 ± 64.24	82.09 ± 52.78	<0.001
	After therapy	42.76 ± 52.96	45.95 ± 49.13	0.855
	△CRP	56.10 ± 60.76	36.18 ± 46.19	<0.001

Neutrophil (%)	Before therapy	63.10 ± 16.06	60.57 ± 45.41	0.052
	After therapy	45.06 ± 15.51	37.99 ± 15.22	<0.001
	△Neutrophils	18.14 ± 15.19	22.54 ± 15.58	0.02

Data are expressed as mean ± *SD*. IVIG = intravenous immunoglobulin; WBC = white blood cells; CRP = C-reactive protein; before therapy: value 1 d before IVIG treatment; after therapy: values 24 hours after IVIG treatment. △: the mean differences in laboratory data were calculated using the following equation: value 1 d before IVIG treatment minus values 24 hours after IVIG treatment.

**Table 3:** Multivariable logistic regression analyses for prediction of IVIG resistance in the data.

Predictor	Logistic coefficient (*β*)	SE	OR	95% CI	*P* value
CRP>100 mg/L	0.66	0.18	1.94	1.36–2.78	<0.001
CALs before therapy	0.74	0.19	2.10	1.46–3.03	<0.001
Intercept	−2.47	0.16			

CALs, coronary artery lesions; IVIG = intravenous immunoglobulin; CRP, C-reactive protein; CI, confidence interval; SE, standard error.

**Table 4:** Coronary artery lesions in the two groups.

Follow-up time	Group 1 (*n* = 141)				Group 2 (*n* = 1140)				*X* ^*2*^	*P* value
	Dilation	SCAA	MCAA	GCAA	Dilation	SCAA	MCAA	GCAA	CALs	CALs
Initial echo at diagnosis	35 (24.8)	17 (12.1)	5 (3.5)	4 (2.8)	210 (18.4)	45 (3.9)	15 (1.3)	8 (0.7)	22.975	<0.001
1 month	25 (17.7)	11 (7.8)	5 (3.5)	6 (4.3)	119 (10.4)	22 (1.9)	11 (1.0)	7 (0.6)	54.217	<0.001
3 months	12 (8.5)	6 (4.3)	4 (2.8)	6 (4.3)	45 (3.9)	10 (0.9)	7 (0.6)	8 (0.7)	33.422	<0.001
6 months	7 (5.0)	5 (3.5)	5 (3.5)	3 (2.1)	13 (1.1)	11 (1.0)	6 (0.5)	6 (0.5)	36.494	<0.001
12 months	4 (2.8)	5 (3.5)	3 (2.1)	2 (1.4)	12 (1.1)	4 (0.4)	3 (0.3)	6 (0.5)	25.441	<0.001

Data are expressed as numbers (percentage). SCAA, small coronary artery aneurysm; MCAA, medium coronary artery aneurysm; GCAA, giant coronary artery aneurysm; CALs, coronary artery lesions = dilation + SCAA + MCAA + GCAA.

## Data Availability

The data that support the findings of this study are available from the corresponding author upon reasonable request.
